# Clinical Implications of the Localization and Morphological Variability of the Mental Foramen—A Systematic Review

**DOI:** 10.3390/diagnostics16050779

**Published:** 2026-03-05

**Authors:** Mariola Krzykawska-Krupska, Janusz Pach, Piotr Regulski, Jacek Tomczyk, Izabela Strużycka, Kazimierz Szopiński, Katarzyna Osipowicz, Anna Pogorzelska

**Affiliations:** 1 Digital Imaging and Virtual Reality Laboratory, Department of Dental and Maxillofacial Radiology, Medical University of Warsaw, ul. Binieckiego St. 6, 02-097 Warsaw, Poland; 2 Institute of Biological Sciences, Cardinal Stefan Wyszyński University, Wóycickiego St. 1/3, 01-938 Warsaw, Poland; 3 Department of Comprehensive Dentistry, Medical University of Warsaw, Binieckiego St. 6, 02-097 Warsaw, Poland; 4 Department of Dentomaxillofacial Radiology, Faculty of Dental Medicine, Medical University of Warsaw, Binieckiego St. 6, 02-097 Warsaw, Poland; 5 Department of Dermatology and Venereology, Infant Jesus Clinical Hospital, Koszykowa St. 82a, 02-008 Warsaw, Poland; 6 Faculty of Health Sciences, University of Kalisz, Nowy Świat St. 4, 62-800 Kalisz, Poland

**Keywords:** mental foramen, anatomical variation, cone-beam computed tomography, mental nerve, diagnostic imaging

## Abstract

**Background**: The mental foramen is a key anatomical structure of the mandible through which the mental nerve and accompanying vessels emerge. Accurate knowledge of its location and morphology is essential for safe dental and surgical procedures in the anterior mandible. **Objective**: This study was conducted as a systematic review to summarize current evidence on the morphology, localization, and anatomical variants of the mental foramen and their clinical relevance. **Methods**: The PubMed and Google Scholar databases were searched for studies published between 2015 and 2025 in accordance with current systematic review guidelines. Cone-beam computed tomography (CBCT) studies and anthropological investigations assessing the position, dimensions, and anatomical variants of the mental foramen were included. **Results**: Thirty-five studies (30 CBCT-based and 5 anthropological) comprising a total of 6240 mandibles or patients were analyzed qualitatively. Considerable variability was observed in the horizontal and vertical position of the mental foramen in relation to mandibular borders and dental landmarks. Anatomical variations included differences in size and shape, the presence of unilateral or bilateral accessory mental foramina, and rare cases of unilateral or bilateral absence of the foramen. **Conclusions**: The synthesis of recent CBCT and anthropological data across diverse populations highlights clinically relevant patterns of variability. This study identifies key positional patterns and variants of the mental foramen, which can inform clinical planning and help reduce the risk of mental nerve injury.

## 1. Introduction

The mental foramen (foramen mentale) is a key anatomical structure of the mandibular body [1]. It constitutes the opening through which the mental nerve and accompanying vessels emerge, supplying sensation to the lower lip, oral mucosa, and gingiva. Understanding its localization, morphology, and potential anatomical variations is crucial in oral surgery, implantology, endodontics, and radiological diagnostics [2]. Precise identification of the mental foramen is indispensable for safe planning of invasive procedures in the anterior mandible, helping minimize neurological complications arising from mental nerve injury, such as paresthesia [3,4,5,6,7].

Individual and population-based variability in the location and number of mental foramina has been reported in numerous radiological and clinical studies [3,4]. Previous reviews often focused on single populations or lacked the most recent CBCT data published after 2019, limiting the ability to formulate generalized clinical conclusions. Existing systematic reviews and meta-analyses typically address only selected aspects (e.g., CBCT imaging or the prevalence of accessory foramina) and cover literature only up to approximately 2020, further justifying the need for an updated and expanded analysis.

The present study is based on the latest international literature and highlights the importance of considering the position and potential duplications or absence of the mental foramen during surgical procedures in the anterior mandible. Morphometric data from imaging and anthropological research are integrated with their practical implications for clinical decision-making across various dental specialties.

The aim of this review was to summarize current knowledge regarding mental foramen morphology, its location in relation to key anatomical landmarks, its associated anomalies, and the clinical relevance of these features for dental and surgical practice. The study was conducted as a literature review in accordance with PRISMA 2020 guidelines [5].

Compared to recent systematic reviews published between 2019 and 2020, and incorporating the most recent studies published from 2020 to 2025, this review provides several novel contributions. First, it includes CBCT-based studies from a broader range of populations worldwide, rather than focusing on single populations or limited regions. Second, it systematically compiles data on rare anatomical variants, such as accessory mental foramina, multiple foramina, and cases of absence of the mental foramen, which were not comprehensively addressed in earlier reviews. Third, it integrates both vertical and horizontal positional data of the mental foramen relative to teeth and mandibular borders, enhancing clinical applicability. Finally, the review translates these morphometric findings into practical guidance for implant placement, endodontic procedures, and surgical planning, highlighting “safety zones” for individualized patient care. Collectively, by including the most up-to-date evidence and a wider global perspective, these aspects fill important gaps in the literature and provide a more comprehensive understanding of mental foramen anatomy across diverse populations.

## 2. Materials and Methods

### 2.1. Search Strategy and Data Extraction

The PubMed and Google Scholar databases were searched for studies published from 2015 to 2025 using the keywords “foramen mentale” and “radiology” and the filter “full text”. As a consequence, grey literature (theses, conference proceedings, and other non-indexed reports) was not systematically searched. A total of 1101 publications were identified, of which 946 were excluded after abstract screening. Studies not directly related to clinical investigations and those examining foramina other than the mental foramen were excluded. Publications that were repeated in both databases were excluded too. The remaining 155 full-text articles were assessed for information concerning localization, shape, and the presence or absence of accessory foramina.

Study selection and data extraction were performed independently by two reviewers to ensure methodological rigor and reduce potential bias. Each reviewer screened titles, abstracts, and full texts according to the predefined eligibility criteria. Any discrepancies between the two reviewers were resolved through discussion, and when consensus could not be reached, a third reviewer was consulted. This approach ensured consistency and reliability in the inclusion of studies and the extraction of relevant data.

### 2.2. Eligibility Criteria

**Figure 1:**
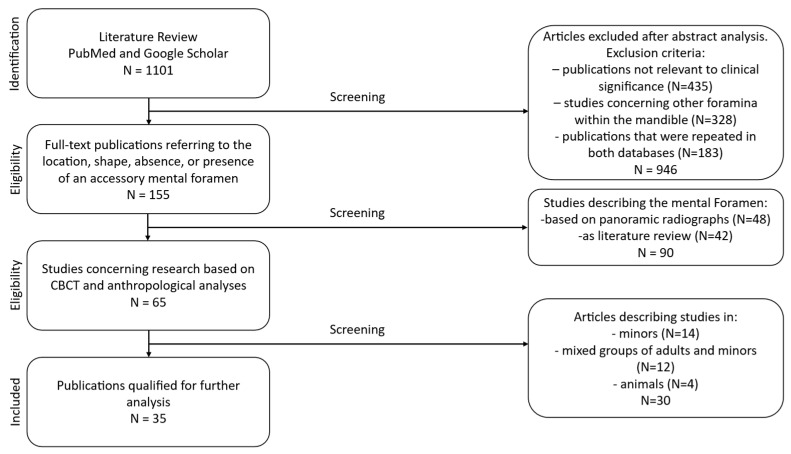
PRISMA flowchart.

Inclusion criteria comprised CBCT-based studies and anthropological investigations. Original observational studies on adult patients or skeletal specimens, regardless of sex or ethnicity, were included. Excluded were panoramic radiograph–based studies and literature reviews. Of the 155 full-text articles assessed, 90 were excluded at the full-text stage because they did not meet the predefined inclusion criteria. Sixty-five publications met the initial criteria, and after excluding studies involving minors, studies involving mixed-age populations without separable adult data, and preclinical studies describing the mental foramen in animal models rather than in humans, 35 papers were retained for final analysis. Articles in English and Polish were evaluated. The review followed the PRISMA 2020 framework, including title/abstract screening and full-text assessment. The PRISMA flowchart [Figure 1] presents the selection process, the number of records identified, duplicate removal, screening exclusions, and full-text rejections. Ultimately, 35 studies (30 CBCT and 5 anthropological) involving 6240 mandibles/patients from various ethnic groups were included.

### 2.3. Risk of Bias and Data Synthesis

Due to substantial heterogeneity in measurement protocols and reported parameters, which precluded meaningful data harmonization, a quantitative meta-analysis was not performed; therefore, a qualitative synthesis was conducted. The PRISMA 2020 checklist accompanies this review in the Supplementary Materials. The methodological quality and risk of bias of the included studies were assessed using the Joanna Briggs Institute (JBI) Critical Appraisal Tools (2025 version), applying the checklist for analytical cross-sectional studies and, where appropriate, the checklist for case reports. The results of the risk of bias assessment are presented in Supplementary Tables S1 and S2. Overall, most studies were judged to be of low to moderate risk of bias.

## 3. Results

The distribution of studies by methodology, population characteristics, and key morphometric parameters is presented in Table 1. Morphometric values for the analyzed variables are summarized in Table 2.

Across the included studies, the mental foramen showed variation in horizontal and vertical position, morphology, and the presence of anatomical variants, as summarized in Table 1 and Table 2.

The mental foramen was most frequently located horizontally between the first and second premolars (position III according to the Tebo and Telford classification), with alignment with the second premolar (position IV) reported more often in selected populations, including Indian, Chilean, and Sudanese cohorts. Vertically, the mental foramen was predominantly positioned below the root apex line across most CBCT and anthropological studies, regardless of population. Morphologically, an oval shape was reported more commonly than round or irregular forms, while anatomical variants such as accessory mental foramina were uncommon and typically reported in a minority of cases. Rare findings, including multiple or absent mental foramina, were limited to isolated case reports.

Although a formal meta-analysis was not feasible due to substantial heterogeneity in measurement protocols, reporting formats, and units of analysis (patients, mandibles, or sides), an alternative quantitative synthesis was performed. Specifically, for studies reporting extractable prevalence data, horizontal mental foramen positions were mapped to the Tebo and Telford classification, and study-level prevalences were summarized using medians (Table 3). This approach allows quantitative comparison of the most commonly reported positions, such as position III and position IV.

**Figure 2:**
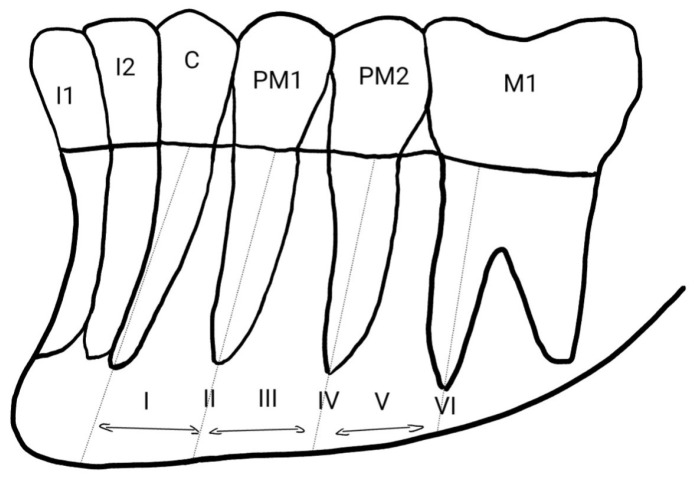
Horizontal position of MF according to Zmysłowska-Polakowska.

To determine the horizontal position of the mental foramen relative to the mandibular dentition, the Tebo and Telford classification is commonly employed. According to this system, the foramen may be located in six positions: mesial to the first premolar (I); in line with the first premolar apex (II); between the first and second premolar roots (III); in line with the second premolar roots (IV); between the second premolar and first molar roots (V); or in line with the mesial root of the first molar (VI) [Figure 2]. Most researchers found the foramen in position III—between the first and second premolars [4,9,10,11,12].

**Figure 3:**
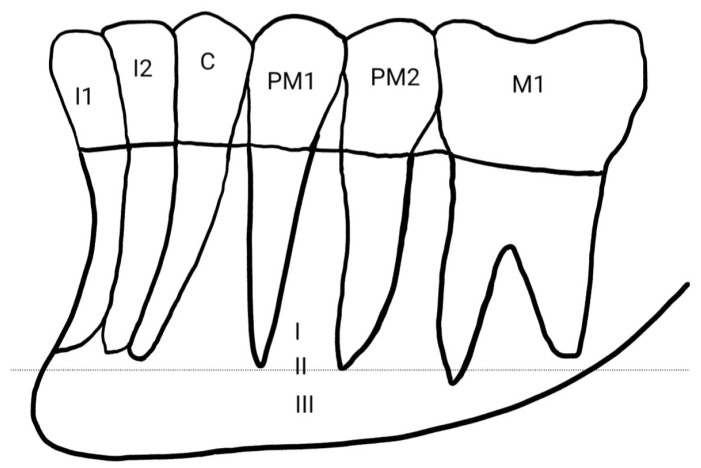
Vertical position of MF according to Zmysłowska-Polakowska.

The vertical position classification proposed by Zmysłowska-Polakowska et al. identifies three positions: above the apical line (I), in line with the apices (II), and below the apical line (III) [Figure 3]. This latter location was most frequently reported in the other publications analyzed [8,18].

Knowledge of the precise location of the mental foramen is also crucial for effective local anesthesia delivery, influencing treatment success and minimizing complications during procedures such as endodontic therapy, apical surgery, periodontal operations, and extractions [8,19]. CBCT provides highly accurate localization [7], whereas panoramic radiographs may fail to reveal of this anatomical structure [16].

One anomaly is the presence of an accessory mental foramen, which typically appears as a small opening near the main foramen, usually located distally and connected to the mandibular canal [11]. A foramen not connected to the canal is referred to as a nutrient foramen [20]. Accessory foramina may occur unilaterally [21] or bilaterally [20]. Cases reporting two accessory foramina on the same side have also been documented [16]. Those connected to the mandibular canal contain neurovascular bundles, and their injury can cause complications such as lower lip numbness [1].

A rare (ranged from 0.02–0.47%) anomaly involves unilateral [22] or bilateral absence of the mental foramen [17]. Cases combining absence on one side and duplication on the other have also been reported [22].

Authors also observed differences in size and shape related to sex and ethnicity, describing the foramen as round, oval, or irregular [3,10,11,23,24].

## 4. Discussion

### 4.1. Position of the Mental Foramen in Relation to the Superior and Inferior Border of the Mandible

Studies conducted on Palestinian, Nepalese, Iranian and Vietnamese populations have shown that the mean distance from the mental foramen to the inferior border of the mandible is greater than its distance to the alveolar crest [6,7,19,25]. In contrast, Ahmed et al., examining a Saudi Arabian population, demonstrated that the mental foramen was located closer to the inferior border of the mandible in both women and men. Their study also showed that the greatest distance between the mental foramen and the inferior border occurred in individuals over 56 years of age, whereas the greatest distance from the alveolar crest was found in participants aged 26–40 years [24].

These discrepancies may reflect a combination of biological and methodological factors. Age-related alveolar bone resorption and tooth loss lead to progressive reduction in the distance between the mental foramen and the superior mandibular border, whereas the distance to the inferior border remains relatively stable. However, population-specific craniofacial morphology, differences in dental status of study samples, and variations in CBCT acquisition protocols and landmark definitions may also contribute substantially to the observed variability. This suggests that normative reference values derived from one population may not be directly transferable to others, underscoring the importance of individualized radiological assessment.

### 4.2. Position of the Mental Foramen in Relation to Tooth Groups

Many authors assess the position of the mental foramen in relation to tooth groups using the classification proposed by Tebo and Telford [4,8,18]. Other researchers report its location with reference to adjacent tooth groups [6,9,25,26]. Studies carried out in Polish [8], Brazilian [27], Syrian [18], Italian [26] and Saudi [3,28,29] populations most commonly found the mental foramen located between the first and second premolars. Similar results were obtained by Reda et al. in another Italian population [13]. Comparable results were also reported by Oliveira et al. in their study of the Brazilian population [30]. This location was consistent on both sides of the mandible.

Different conclusions were reported by researchers from Chile [12], India [9,10], Jordan [4], Sudan [25] and Palestine [6], who most frequently observed the foramen aligned with the long axis of the second premolar. In these studies, the frequency of this location was similar on the left and right sides.

Studies in a Romanian population showed that the mental foramen was most commonly located below the second premolar on the left side, while on the right side it was most often positioned between the premolars [14]. Subramanian et al. demonstrated in their study of the Zambian population that the position of the mental foramen, on both the right and left sides, was most frequently located between the second premolar and the first molar [31].

These interpopulation differences likely reflect complex interactions between genetic background, mandibular growth patterns, dental arch morphology and functional loading related to diet and mastication. In addition, methodological variation—particularly differences in reference points, classification systems and CBCT acquisition protocols—may partially explain inconsistent findings between studies. Together, these factors highlight the need for population-specific datasets and standardized reporting frameworks in future research.

### 4.3. Vertical Position of the Mental Foramen

According to the classification proposed by Zmysłowska-Polakowska et al., describing the vertical position of the mental foramen, all authors investigating the location of this structure found that it most commonly appears in position III, i.e., below the root apex line [8,18,26].

This relative uniformity suggests that, despite substantial horizontal positional variability, the vertical relationship of the mental foramen to adjacent tooth apices may be more conserved across populations. Nevertheless, advanced alveolar bone resorption, particularly in partially edentulous and edentulous patients, may significantly alter this relationship and should always be evaluated using CBCT.

### 4.4. Shape of the Mental Foramen

Most studies assessing the shape of the mental foramen reported that it is predominantly oval [3,15,21,26]. However, Sheth et al. found that its shape was round in the majority of their study population [9].

These discrepancies may reflect true population-specific morphological variation or differences in image resolution, slice thickness and observer interpretation. From a clinical perspective, shape variability may influence detectability of the foramen and the apparent width of the neurovascular bundle, reinforcing the importance of high-quality three-dimensional imaging in surgical planning.

### 4.5. Presence of Accessory Mental Foramina, Absence of the Mental Foramen and Its Rare Variation

An accessory mental foramen is usually a small opening in the mandibular body that communicates with the mandibular canal and is typically located near the main mental foramen [11]. The reviewed literature includes cases describing unilateral accessory foramina [6,21,26,32], while other authors reported accessory foramina present bilaterally [11,20]. Thomaidi et al. documented a case of two accessory mental foramina on one side [1]. Yovchev et al. described an instance of an accessory foramen on the right side with simultaneous absence of the main foramen on the left [22]. Khaled et al. reported a patient with unilateral absence of the mental foramen on the left side while demonstrating normal sensory function of the lips, gingiva and chin [33]. Lauhr et al. reported a bilateral absence of the mental foramen in the mandible of a Caucasian male [17]. Borghesi et al. described a case of a patient with the presence of five mental foramina [34]. Predoiu et al. reported that in a 28-year-old European female, three mental foramina were present on the right side. On the left side, a single mental foramen was observed, located between the apices of the premolar roots [35].

A particularly rare case involving the mental foramen was described by Tallada et al. In their examination of the mandible of a 31-year-old woman, they identified the presence of an accessory mental foramen on the right side, located lingually. Both foramina (the buccal and the lingual) were anatomically connected [36].

This configuration challenges the conventional assumption of exclusively buccal emergence of the mental nerve and underscores the necessity of multiplanar CBCT evaluation rather than reliance on two-dimensional imaging alone.

These findings emphasize that reliance on expected anatomical patterns alone may lead to iatrogenic nerve injury, particularly during implant placement, apical surgery or flap elevation. Comprehensive CBCT assessment is therefore essential to identify accessory canals, bifurcations and atypical exit points of the mental nerve.

The present review enabled the compilation of data from various regions of the world, revealing both recurrent topographic patterns of the mental foramen and significant population-specific differences. This represents added value compared with earlier studies focused on single populations or older specimen series.

Specifically, unlike earlier narrative reviews or single-population analyses, the present study integrates CBCT-based morphometric data from multiple geographic regions, systematically summarizes rare anatomical variants, and explicitly links anatomical variability with clinical risk management strategies. In doing so, it addresses gaps in the literature regarding the global variability of mental foramen topography and its relevance to contemporary implantology and oral surgery.

### 4.6. Limitations

This review has several limitations. The included studies differed substantially in terms of CBCT protocols, exposure parameters, definitions of anatomical landmarks, and population characteristics, making a quantitative meta-analysis impossible. Not all studies reported sampling strategies, calibration procedures, or measurement conditions in sufficient detail, which increases the risk of systematic bias.

Panoramic radiograph studies were deliberately excluded to obtain the most precise and comparable morphometric data regarding the mental foramen. CBCT allows three-dimensional assessment of mandibular structures without geometric distortions or superimposition of tissues, which are inherent limitations of panoramic imaging. However, we recognize that panoramic studies constitute a substantial portion of the literature and may provide useful comparative information. Their exclusion may introduce selection bias and affect the overall representativeness of the review.

Limiting the review to full-text articles published in English and Polish may also have resulted in language bias, excluding relevant research published in other languages. Additionally, publication bias should be considered, as studies reporting rare anatomical variants or statistically significant findings may be overrepresented. Finally, applying a full-text availability restriction and not systematically searching grey literature (theses, conference proceedings, and other non-indexed reports) may have led to missing relevant evidence.

These factors should be kept in mind when interpreting the results and generalizing the conclusions. Despite these limitations, we believe the review provides a comprehensive synthesis of CBCT-based morphometric and topographic data on the mental foramen, including rare anatomical variants, which is valuable for both clinical and anthropological applications.

### 4.7. Clinical Implications

The collected data confirm that in most studied populations, the mental foramen is most commonly located between and below the apices of the first and second premolars or below the second premolar, below the root apex line. This information is essential when planning implant placement, root apex resections and mucoperiosteal flap design in the anterior mandible. Considering anatomical variations—such as the presence of an anterior loop, accessory foramina or positioning closer to the lower mandibular border—allows for establishing individualized “safety zones” for each patient based on CBCT assessment, thus minimizing the risk of injury to the mental nerve.

However, the substantial interindividual and interpopulation variability observed across studies suggests that fixed “safe distances” based solely on average anatomical values may be insufficient. Instead, individualized CBCT-based risk assessment should be used to define patient-specific safety margins.

Based on the analyzed studies, several parameters should be routinely included in CBCT reports: vertical and horizontal position of the mental foramen, distance to adjacent root apices, relationship to the mandibular borders, and presence of anatomical variants (accessory foramina, anterior loop). Standardizing these descriptions would facilitate comparison between centers and improve surgical and implantological safety.

### 4.8. Future Research Directions

The findings indicate several areas requiring further investigation.

First, there is a lack of large, prospective CBCT studies that simultaneously account for age, sex, degree of alveolar bone resorption, dental status and ethnic background as potential determinants of mental foramen position.

Second, studies combining morphometric analysis of the mental foramen with treatment outcomes (e.g., rates of neurological complications after implant placement or apical surgery) are needed to verify the clinical value of proposed “safety zones”.

Another promising direction is the use of machine learning and automated segmentation in CBCT imaging to detect mental foramina and their anatomical variants. Developing and validating such tools could support treatment planning, particularly in patients with atypical morphology or advanced bone loss.

Finally, large, well-characterized cranial collections from diverse geographic regions should be included in anthropological studies to better understand population and evolutionary determinants of mental foramen variability and their relevance to contemporary clinical practice.

## 5. Conclusions

This systematic review provides a comprehensive summary of the anatomical variations in the mental foramen across different populations. Our findings confirm that the mental foramen may vary in shape (oval, round, or irregular), vertical position (closer to the alveolar crest or inferior border), and horizontal location (ranging from mesial to the first premolar to the mesial root of the first molar). Rare anomalies, such as unilateral or bilateral accessory foramina or absence of the foramen, were also identified.

The MF most commonly corresponded to position III (between the first and second premolars), with a median prevalence of 56% and a wide interpopulation range of 26–92%. Position IV was also frequently reported (median 51.3%). Vertically, the MF was predominantly located below the root apex line, with frequencies reaching 48–92% in CBCT-based studies.

Morphometric data showed that the distance between the MF and the inferior mandibular border ranged from 10 to 16 mm, while the anterior loop length reached up to 7.5 mm. Accessory mental foramina were observed in 1–12% of cases, whereas complete absence of the MF was rare (approximately 0.02–0.47%).

The compiled data offer practical guidance for clinicians in oral surgery, implantology, orthodontics, and endodontics, emphasizing the importance of assessing the position of the mental foramen to minimize the risk of mental nerve injury and related post-operative complications. This review serves as a basis for standardizing CBCT reporting and developing population-specific recommendations, supporting individualized clinical planning for implant placement, endodontic procedures, and surgical interventions in the anterior mandible.

## Figures and Tables

**Table 1:** Characteristics of selected studies included in the review.

Study ID	Country/ Population	Sample Size (Mandibles/Patients)	Method	MF Horizontal Position	MF Vertical Position	Variants Reported	Key Finding
Abu-Ta’a et al., 2023 [6]	Palestine	106 cases	CBCT	III (PM1–PM2) 49%	Below apex 48%	Accessory MF rare	Predominant location of MF between the first and second premolars, observed in nearly half of the studied cases
Mallahi et al., 2024 [7]	Iran	355 cases	CBCT	II–III	Below apex	Anterior loop	MF most commonly located inferiorly, with frequent identification of an anterior loop in this population
Zmysłowska- Polakowska et al., 2019 [8]	Poland	201 cases	CBCT	III 56%	Below apex 54%	—	Type III horizontal position was the most frequent pattern in the Polish population, exceeding half of the examined cases
Sheth et al., 2021 [9]	India	475 cases	CBCT	III 71%	Below apex 66%	—	High predominance of type III horizontal position in the Indian population, observed in more than two-thirds of cases
Srivastava et al., 2024 [10]	India	250 cases	CBCT	PM2 62%	Below apex 62%	Anterior loop (long)	Anterior loop length reached up to 6–7 mm, representing a clinically relevant extension of the mental nerve
Barbosa et al., 2024 [11]	Brazil	250 cases	CBCT	III 58%	Below apex 58%	Accessory MF	Accessory mental foramina were detected in a notable proportion of cases, indicating a non-negligible prevalence in this population
Guzmán et al., 2024 [12]	Chile	342 cases	CBCT	PM2 62%	Below apex 62%	—	Alignment of the MF with the second premolar represented the dominant horizontal position in this Chilean subpopulation
Reda et al., 2022 [13]	Italy	492 cases	CBCT	III 92%	Below apex 92%	—	Symmetrical positioning of MF was consistently observed on both sides of the mandible
Gherghiță et al., 2021 [14]	Romania	40 cases	CBCT	Left: PM2/41% Right: PM1–PM2 56%	Below apex	—	Distinct side-related differences in horizontal MF position were identified between the left and right mandible
Shashidhar et al., 2019 [15]	India	180 cases	Anthropometry	IV 88%	Below apex	—	The mental foramen was most frequently positioned posterior to the second premolar, with no side-related differences observed
Mohebiniya et al., 2024 [16] (case report)	Iran	1 case	CBCT	Variable	—	Multiple MF	Rare anatomical variation characterized by bilateral duplication of MF identified on CBCT imaging
Lauhr et al., 2015 [17] (case report)	Caucasus	1 case	CBCT	—	—	—	Extremely rare anatomical finding of bilateral absence of MF documented in a living subject

Footnote: MF position: II (in line with the first premolar apex), III (between the first and second premolar roots), IV (in line with the second premolar roots); PM1—first premolar, PM2—second premolar.

**Table 2:** Morphometric values.

Parameter	Reported Range	Reported Mean ± SD (Where Available)	Studies Reporting Mean (n)	Notes
Distance MF–Inferior Border	10–16 mm	11.83 ± 1.83 mm, 11.54 mm; 13.52 mm	3	Often greater in older patients due to alveolar bone resorption
Distance MF–Alveolar Crest	7–12 mm	not reported	0	Greatly reduced in edentulous mandibles
Horizontal position	Mostly position III (PM1–PM2)	not reported	0	With exceptions in Sudanese, Indian, Chilean populations
Vertical position	Predominantly below root apices	not reported	0	Type III (Zmysłowska-Polakowska)
MF shape	Oval > Round > Irregular	not reported	0	Shape correlated with sex in some populations
Accessory MF	1–12%	not reported	0	Most commonly located distally to main MF
Anterior loop length	1–7.5 mm	Right: 6.43 ± 2.17 mm; Left: 6.56 ± 1.81 mm	1	Must be evaluated preoperatively

Footnote: III (MF position between the first and second premolar roots); PM1—first premolar, PM2—second premolar.

**Table 3:** Reported prevalence (%) of horizontal mental foramen positions according to the Tebo and Telford classification across included studies.

Study	I	II	III	IV	V	VI
Abu-Ta’a et al., 2023 [6]	NR	NR	49	NR	6/5	NR
Mallahi et al., 2024 [7]	NR	NR	NR	NR	NR	NR
Zmysłowska-Polakowska et al., 2019 [8] (right/left site)	0/0	4.5/5.0	58.7/55.4	34.4/31.3	4.5/7.9	0/0
Sheth et al., 2021 [9] (right/left site)	0/0	6/10	26/14	62/71	6/5	0/0
Srivastava et al., 2024 [10]	0	1.0	27.2	61.6	9.8	0.4
Barbosa et al., 2024 [11]	NR	NR	NR	NR	NR	NR
Guzmán et al., 2024 [12]	NR	9.5	62.0	25.5	3.0	NR
Reda et al., 2022 [13]	NR	8	92	NR	NR	NR
Gherghiță et al., 2021 [14]	NR	NR	56	41	NR	NR
Shashidhar et al., 2019 [15]	NR	NR	NR	88	NR	NR
Median (%)	0.0	8.8	56.0	51.3	6.4	0.4

Footnote: MF Position according the Tebo and Telford classification: I—mesial to the first premolar, II—in line with the first premolar apex, III—between the first and second premolar roots, IV—in line with the second premolar roots, V—between the second premolar and first molar roots, VI—in line with the mesial root of the first molar; NR = not reported.

## Data Availability

No new data were created or analyzed in this study.

## Supplementary Materials

The following supporting information can be downloaded at: https://www.mdpi.com/article/10.3390/diagnostics16050779/s1, Table S1: Risk of bias appraisal of included observational studies using the JBI Critical Appraisal Checklist for Analytical Cross-Sectional Studies. Table S2: Risk of bias appraisal of included case reports using the JBI Critical Appraisal Checklist for Case Reports. Table S3: PRISMA 2020 Checklist.
