# Supplementary material for: Clinical Implications of the Localization and Morphological Variability of the Mental Foramen—A Systematic Review
Source: Diagnostics (Basel). 2026 Mar 5;16(5):779. doi: 10.3390/diagnostics16050779 (PMC12984819; doi:10.3390/diagnostics16050779)
Supplement: Supplementary file 1 [file diagnostics-16-00779-s001.zip › diagnostics-4118670-supplementary.pdf]

Table S1. Risk of bias appraisal of included observational studies using the JBI Critical Appraisal Checklist for Analytical Cross-Sectional Studies.

Study	Method	Q1	Q2	Q3	Q4	Q5	Q6	Q7	Q8	Overall risk of bias
Abu-Ta’a et al. 2023	CBCT	Y	Y	Y	Y	U	U	U	Y	Low
Mallahi et al. 2024	CBCT	Y	Y	Y	Y	Y	Y	U	Y	Low
Zmysłowska-Polakowska et al. 2019	CBCT	Y	Y	Y	Y	U	U	U	Y	Moderate
Sheth et al. 2021	CBCT	Y	Y	Y	Y	Y	Y	Y	Y	Low
Srivastava et al. 2024	CBCT	Y	Y	Y	Y	Y	Y	U	Y	Low
Barbosa et al. 2024	CBCT	Y	Y	Y	Y	Y	Y	Y	Y	Low
Guzmán et al. 2024	CBCT	Y	Y	Y	Y	U	U	U	Y	Moderate
Reda et al. 2022	CBCT	Y	Y	Y	Y	U	U	U	Y	Moderate
Gherghiță et al. 2021	CBCT	Y	Y	Y	Y	U	U	U	Y	Moderate
Shashidhar et al. 2019	Anthropometry	Y	Y	Y	Y	U	U	U	Y	Moderate

Footnote: Q1—Inclusion criteria clearly defined; Q2—Study subjects and setting described in detail; Q3—Exposure measured in a valid and reliable way; Q4—Objective, standard criteria used for measurement of the condition; Q5—Confounding factors identified; Q6—Strategies to deal with confounding factors stated; Q7—Outcomes measured in a valid and reliable way; Q8—Appropriate statistical analysis used. Ratings: Y = Yes; N = No; U = Unclear; NA = Not applicable.

Table S2. Risk of bias appraisal of included case reports using the JBI Critical Appraisal Checklist for Case Reports.

Study	Method	Q1	Q2	Q3	Q4	Q5	Q6	Q7	Q8	Overall risk of bias
Mohebiniya et al. 2024	CBCT	Y	U	Y	Y	NA	NA	NA	Y	Low
Lauhr et al. 2015	CBCT	Y	U	Y	Y	NA	NA	NA	Y	Low

Footnote: Q1—Patient demographic characteristics described; Q2—Patient history clearly described and presented as a timeline; Q3—Clinical condition on presentation described; Q4—Diagnostic tests/assessment methods and results described; Q5—Intervention(s) or treatment procedure(s) described; Q6—Post-intervention clinical condition described; Q7—Adverse/unanticipated events described; Q8—Takeaway lessons provided. Ratings: Y = Yes; N = No; U = Unclear; NA = Not applicable.
